# Fornix Reconstruction in Cicatricial Ocular Surface Disease: A Narrative Review of Graft Materials, Surgical Techniques, and Medical Management

**DOI:** 10.7759/cureus.116613

**Published:** 2026-09-20

**Authors:** Muhammad Awan, Cuneyt Ozkardes, Zhenyang Zhao, Mohammad Soleimani

**Affiliations:** 1 Research, Alabama College of Osteopathic Medicine, Dothan, USA; 2 Ophthalmology, Emory University School of Medicine, Atlanta, USA; 3 Ophthalmology, University of Southern California Keck School of Medicine, Los Angeles, USA

**Keywords:** amniotic membrane transplantation, cicatricial disease, fornix reconstruction, mitomycin c (mmc), mucous membrane pemphigoid, oral mucosa graft, stevens-johnson syndrome, symblepharon

## Abstract

Conjunctival fornix reconstruction is performed to restore fornix depth following symblepharon formation or conjunctival contracture, thereby improving ocular surface function, the tear reservoir, and ocular motility. In severe cicatricial disease, it also serves as the foundational first step in staged ocular surface rehabilitation. However, long-term surgical success is often limited by recurrent fibrosis from chronic inflammation. This narrative review examines the graft materials, surgical techniques, and medical management used in fornix reconstruction. A structured search of PubMed/MEDLINE from database inception through July 2026 was conducted. Relevant literature included original research, case series, prior reviews, and clinical practice guidelines. Twenty-one publications met the eligibility criteria, 15 of which reported fornix-specific outcomes. Clinical findings consistently varied with disease severity. Amniotic membrane transplantation (AMT) alone produced favorable results in mild disease but substantially lower rates of complete success in advanced cicatrization. The addition of oral mucosal or conjunctival grafts improved outcomes in severe cases. Multimodal approaches combining cicatrix lysis, mitomycin C, mucosal grafting, and AMT achieved high rates of successful reconstruction in advanced disease. AMT combined with intraoperative mitomycin C also showed benefit in eyes with previously failed reconstruction. In contrast, outcomes were less durable in late-stage ocular cicatricial pemphigoid, with recurrent symblepharon on follow-up. Cultivated oral mucosal epithelial transplantation demonstrated encouraging intermediate- and long-term success, while cultivated limbal epithelial sheets appeared less effective in patients with more advanced disease. Because published definitions of success differ, reported rates are not directly comparable. Emerging approaches, such as tissue-engineered epithelial substitutes and contact lens-based stem cell delivery, may address limitations of current techniques but have not been established for fornix reconstruction. Overall, the evidence base consists predominantly of small, single-center, non-comparative case series. Future prospective multicenter studies using standardized severity grading, clearly defined success criteria, and longer follow-up are needed to better compare the effectiveness and durability of current reconstruction approaches.

## Introduction and background

The conjunctival fornices are deep recesses formed where the bulbar conjunctiva reflects onto the palpebral conjunctiva. They serve as reservoirs for the tear film, provide the redundant conjunctiva necessary for a full range of ocular motility, and allow for complete eyelid closure [1]. Fornix shortening can lead to symblepharon formation, resulting in tear-film instability, patient discomfort, and restriction of ocular motility. Severe cases may require fornix reconstruction to restore normal ocular surface health and vision [2,3].

Fibrosis and contracture of the subconjunctival tissue are driven by chronic inflammation and aberrant wound healing [4]. Subconjunctival fibrosis is implicated in both the initial shortening and the recurrence that limits durability after surgery. However, the relative contributions of residual scar, surgical trauma, and continuing disease activity to postoperative recurrence have not been separately quantified. For this reason, reconstructive strategies described in the literature have generally combined replacement of the deficient lining with measures that are intended to limit postoperative scarring, instead of relying on tissue replacement alone [3,5,6]. In ocular surface disease, the primary aims of fornix reconstruction are to restore ocular motility, re-establish the tear reservoir and a stable epithelium, resolve cicatricial lid malposition, and prepare the surface for any future corneal or limbal reconstruction.

Reported prognostic associations are broadly consistent with the underlying pathophysiology, although all of them derive from observational data and none has been validated prospectively. Better outcomes are generally associated with a lower grade of symblepharon, traumatic or non-autoimmune etiology, intraoperative use of adjunctive antifibrotic agents, such as mitomycin C (MMC) or 5-fluorouracil (5-FU), and a non-inflamed surface at the time of surgery. Worse outcomes appear to be associated with canthal involvement and autoimmune etiologies (e.g., ocular cicatricial pemphigoid), both of which indicate more sustained scarring and inflammation working against the repair [2,3,7]. However, the largest series of this review found no significant association between the etiology of symblepharon and surgical outcome, suggesting that etiology may serve as a prognostic signal but is not an independently validated predictor of surgical outcome [3]. In autoimmune cicatrizing disease (e.g., mucous membrane pemphigoid), surgery alone may not be sufficient. Systemic inflammation should be adequately controlled prior to reconstruction, and surgery should therefore be deferred until the disease is quiescent.

This narrative review focuses on fornix reconstruction of the ocular surface and is organized around graft materials, surgical techniques, and medical management studied in the fornix-specific literature. Although reconstruction of the contracted anophthalmic socket shares many materials and techniques with fornix reconstruction, it has different objectives and is therefore not the primary focus of this review.

## Review

Methods

A narrative review was conducted using PubMed/MEDLINE to identify original research, case series, systematic reviews, and clinical practice guidelines from database inception to July 2026. The search was limited to English-language publications. No restriction was placed on the year of publication because several of the techniques described were first reported in the 1990s and early 2000s, and the original reports remain the primary source for them. Search terms comprised free-text terms relating to fornical anatomy and reconstructive intervention:

("fornix reconstruction" OR "conjunctival fornix" OR "fornices" OR "symblepharon").

AND

("amniotic membrane" OR "oral mucosa" OR "oral mucosal" OR "mucous membrane graft" OR "mucous membrane grafting" OR "conjunctival autograft" OR "mitomycin" OR "cultivated" OR "reconstruction" OR "surgical" OR "surgery" OR "graft").

Terms were entered as free text without a MeSH-only restriction or a publication-type filter. The reference lists of all included articles and of relevant prior reviews were hand-searched for further eligible publications.

Publications were included if they met all the following: (1) addressed fornix reconstruction of the ocular surface in cicatricial disease in any age group; (2) reported on at least one of etiology, pathogenesis, severity grading, surgical technique, graft material, adjunctive therapy, immunosuppression, complications, or outcomes; and (3) were original research (case reports and case series, observational studies, and interventional studies), a narrative or systematic review, or a consensus statement.

Publications were excluded if (1) fornix reconstruction of the ocular surface was not the primary focus; (2) fornix-specific outcomes could not be separated from other ocular surface endpoints; (3) reconstruction was performed in an anophthalmic socket rather than an eye with an intact globe, unless the material or technique was directly transferable; (4) the publication was not in English; or (5) the full text could not be obtained.

The search yielded 681 articles. Eligibility was assessed in two stages. The titles and abstracts of the 681 records were first screened for relevance to the topic of the review, and potentially eligible reports then underwent full-text assessment. Screening and full-text assessment were performed by one reviewer, and the final set of included studies was verified by a second author. Reference lists of included articles were subsequently re-screened, which identified four further eligible studies that had been missed at the title and abstract stage. In total, 21 publications met the eligibility criteria. Of these, 15 studies report fornix-specific outcomes. The remaining six inform the review of severity grading, medical management, or mechanism and are discussed in the text.

Additional publications outside these criteria, including studies of anophthalmic sockets and procedures from other surgical fields, are cited when a technique, material, or mechanism is directly applicable to fornix reconstruction. Such evidence is identified as extrapolated data at the point of citation and is not included in the count of eligible publications.

Because this is a narrative review rather than a systematic review, a PRISMA flow diagram and quantitative risk-of-bias assessment were not undertaken. Established risk-of-bias instruments are constructed principally for comparative studies and discriminate poorly among single-arm case series, which constitute all but one of the clinical studies identified in this review. Each included study was instead appraised qualitatively against the following features: study design and direction (prospective or retrospective), consecutive or selected enrollment, sample size, duration of follow-up, and whether a success criterion was prespecified and explicitly defined. These features are reported in a table so that the strength of each result can be weighed directly.

No pooled estimate, meta-analysis, or other quantitative synthesis was performed, and the evidence synthesis is therefore qualitative. All p-values, confidence intervals, and survival estimates reported in this review are reproduced from the primary publications. No statistical testing was undertaken by the authors. When an estimate is based on a small subgroup, the subgroup size is reported alongside the estimate.

Reliance on a single database (PubMed/MEDLINE), restriction to English-language publications, the absence of formal quality grading, and the predominance of small, retrospective, single-center case series are acknowledged limitations of our approach.

Definition of Success in Fornix Reconstruction

A challenge in comparing fornix reconstruction outcomes is the lack of a standardized definition of "success". Studies define success differently in almost every series. Reported endpoints include anatomical fornix depth, the absence of recurrent symblepharon, restoration of ocular motility, stability of the ocular surface epithelium, and the ability to proceed to further reconstructive surgery. These endpoints are related but not interchangeable, and the differences in their definitions can substantially influence comparison of the reported success rates.

For example, Kheirkhah et al. defined complete success as restoration of an anatomically deep fornix without scar or motility restriction and partial success as the focal recurrence of scar tissue. Overall success combined complete and partial success, and therefore their figure of 85.2% overall success includes eyes with residual focal scarring [3,5]. Solomon et al. required the resolution of any preoperative motility restriction for a case to count as a success [2]. Tseng et al. defined success as restoration of a stable, deep fornix without scarring, inflammation, or ocular motility restriction [6]. Other studies used more permissive or function-specific criteria, including tolerance of mild recurrent symblepharon without functional or cosmetic disability, longitudinal assessment of symblepharon and fornix-shortening scores, and the ability of the reconstructed fornix to support a type I keratoprosthesis [7-9].

These differences reflect an important clinical principle that the appropriate measure of success depends on the purpose of reconstruction. When surgery is performed to prepare the eye for subsequent limbal or corneal reconstruction, the key outcome is whether the fornix is sufficiently deep and stable to support the next procedure [6,9]. When scarring causes diplopia due to restriction, restoration of ocular motility may be the focused endpoint [2]. In progressive autoimmune disease, long-term durability may be more informative than the immediate result [8,10]. Hence, throughout the review, the definition of success is reported alongside the outcome if the study specifies it.

Severity Grading and Evidence-Based Surgical Framework

A severity-based classification of symblepharon proposed by Kheirkhah et al. guides surgical decision-making by characterizing the extent of symblepharon and fornix shortening [3]. The system grades three features: the extent of fornix shortening (length), horizontal extent of symblepharon (width), and conjunctival inflammatory activity. Length is graded I-IV according to the distance from the lid margin to the limbus through the symblepharon, with higher grades indicating progressively greater fornix shortening. Grade IV represents nearly complete obliteration of the fornix, as in ankyloblepharon. Width is graded as a, b, or c according to whether the symblepharon spans up to one-third, up to two-thirds, or more than two-thirds of the eyelid length. Inflammatory activity is graded 0 to 3+ according to conjunctival hyperemia. Canthal involvement is recorded separately. In the original series, higher length grades were associated with greater motility restriction and canthal involvement, but not with greater conjunctival inflammatory activity [3].

The prognostic value of the length grade has since been reproduced independently. In a retrospective series of 80 eyes with symblepharon following chemical or thermal burns, patients were reconstructed with allogeneic cultivated limbal epithelial sheets rather than membrane or mucosal grafts. Complete success after the first procedure was 85.0% in grade I/II, 51.5% in grade III, and 22.2% in grade IV disease (p = 0.001, chi-square). On multivariate analysis, recurrence was associated with symblepharon length (p = 0.003), preoperative inflammatory activity (p = 0.016), and postoperative cicatricial entropion or trichiasis (p = 0.038) [11]. The appearance of the same gradient at a second center using a different graft material supports the length grade as a description of the reconstructive problem. Specifically, it reflects how much lining must be replaced rather than being a property of any one technique.

Surgically, the value of the grading lies in the amount of conjunctiva remaining after cicatrix lysis. In Grade I disease, the residual recessed conjunctiva is sufficient to cover the palpebral surface. In Grade II, it covers the tarsus but not the entire palpebral surface. In the original series, both Grade I and II disease were successfully managed with cicatrix lysis, conjunctival recession, and amniotic membrane transplantation (AMT) alone [3]. In Grade III and IV disease, the residual conjunctiva is insufficient to cover the tarsus or is absent altogether after lysis, respectively. These larger defects may therefore require replacement with a graft, such as oral mucosa, conjunctival autograft, or a cultivated epithelial sheet, in addition to AMT [3,5].

Table 1 summarizes an evidence-informed framework for surgical decision-making based on disease severity, underlying etiology, and status of the ocular surface. The table is intended to organize the available evidence rather than serve as a treatment algorithm or standard of care. It summarizes approaches supported primarily by observational studies and expert consensus, and there is currently no randomized evidence directly comparing the approaches listed. Accordingly, the choice of approach should be individualized based on disease activity, tear function, prior surgery, donor tissue availability, and the intended subsequent reconstruction, factors that are not captured by symblepharon grade alone.

**Table 1:** Evidence-informed framework for surgical decision-making in fornix reconstruction This table summarizes approaches consistent with published observational data; it is not an established treatment algorithm. Abbreviations: AMT, amniotic membrane transplantation; COMET, cultivated oral mucosal epithelial transplantation; MMC, mitomycin C; MMP, mucous membrane pemphigoid; OCP, ocular cicatricial pemphigoid; SJS/TEN, Stevens-Johnson syndrome/toxic epidermal necrolysis

Clinical scenario	Approach consistent with available evidence	Supporting evidence
Grade I-II symblepharon (Residual conjunctiva covers the tarsus or entire palpebral surface after lysis)	Cicatrix lysis + conjunctival recession + AMT. Consider intraoperative MMC if the ocular surface is inflamed. Use anchoring sutures selectively rather than routinely.	Retrospective data showed overall success (complete plus partial) in 13/14 grade I eyes and 6/6 grade II eyes treated with lysis + AMT alone. An independent series graded with the same system reported complete success in 85.0% of grade I/II eyes using a cultivated epithelial sheet [11]. Anchoring sutures were associated with lower success rates, likely because they were preferentially used in more difficult cases [3].
Grade III-IV symblepharon (Insufficient or absent conjunctiva to cover the tarsus)	Cicatrix lysis + MMC + tissue grafting. Replace missing lining with an oral mucosal graft or, if suitable donor tissue is available, a conjunctival autograft. Cover bare sclera with AMT and use anchoring sutures to maintain fornix depth.	One series reported 11/11 successes when a mucosal or conjunctival graft was added, compared with 5/7 with lysis + AMT + anchoring sutures alone [3]. A separate prospective series reported 84.4% complete success in 32 grade III-IV eyes [5]. In the same independent series, complete success fell to 51.5% in Grade III and 22.2% in Grade IV disease [11]. These series are single-center and non-comparative.
Severe cicatricial disease with limited donor tissue or bilateral disease	Consider COMET as an alternative source of autologous epithelium, typically as part of a staged reconstruction.	Two small case series reported different outcomes: 79.6% 5-year success probability in 16 eyes [8]; success in 11/19 eyes with partial success in another 5 [9]. There are no direct comparisons with conventional mucosal grafting.
Autoimmune cicatrizing disease (e.g., MMP/OCP)	Delay elective reconstruction until the systemic disease is quiescent and adequately immunosuppressed. Maintain inflammatory control around surgery. Counsel patients on a higher recurrence risk compared to stable disease. Serial subconjunctival 5-FU injection may be considered as an adjunct in progressive cicatrization.	Based primarily on consensus recommendation and expert practice [12]. Two small prospective series of treated, quiescent disease showed acceptable outcomes in very small numbers [5,9]. Advanced, actively treated disease was associated with recurrence over time [10]. A later cohort reported that no MMP eye retained fornix depth at final follow-up, with recurrence documented in 7/10 eyes at a mean of 6.4 months [13]. A retrospective cohort reported less severe symblepharon and fewer mucous membrane graft procedures in eyes receiving serial 5-FU injections [14].
Acute SJS/TEN	During the acute phase, apply AMT to the lid margins, palpebral conjunctiva, and ocular surface. Defer definitive fornix reconstruction until the disease is quiescent.	A prospective series of 10 patients treated during the acute phase showed mild-to-moderate scarring and best-corrected acuity of 20/30 or better [15]. Findings were supported by a subsequent review [16]. This treatment is intended to prevent cicatrization, not to reconstruct a fornix.
Severe aqueous tear deficiency or keratinized ocular surface	Optimize the tear film before reconstruction. Consider deferring or excluding reconstruction in eyes with severe untreated aqueous deficiency.	In one prospective series, all 4 eyes with a preoperative Schirmer value below 5 mm failed [7]. Two later prospective series excluded patients with total keratinization or severe dry eye despite punctal occlusion [5,9].

Graft Materials for Fornix Reconstruction

The choice of graft material is determined by how much tissue must be replaced and by how likely the underlying disease is to undo the repair through recurrent scarring. The materials below are presented in the order in which they are generally recruited as the severity of symblepharon increases.

(I) Amniotic membrane transplantation (AMT): It is the most widely used biological material for fornix reconstruction and the default choice for lower-grade disease. It is valuable in that it directly counters the fibrosis involved in fornix shortening; it is anti-inflammatory, antifibrotic, and anti-angiogenic. Amniotic membrane also promotes epithelial migration and differentiation across the reconstructed fornix, and it suppresses transforming growth factor-β signaling in subconjunctival fibroblasts [17,18]. By resurfacing the bare episclera after cicatrix lysis, AMT provides a scaffold for epithelialization while physically separating the palpebral and bulbar surfaces to help reduce re-adherence [17].

Clinical outcomes suggest that AMT is most effective when sufficient conjunctiva remains after lysis. In a mixed-etiology series, Solomon et al. reported complete success in 12 of 17 eyes (70.6%) treated with AMT and full-thickness lid-anchoring sutures. Their definition of success required both a deep, scar-free fornix at six months or more and resolution of any preoperative motility restriction. The outcomes were influenced by underlying disease, wherein all post-traumatic cases succeeded while partial successes and failures occurred in eyes with autoimmune disease or recurrent pterygium [2]. Jain et al. reported recurrence of symblepharon in eight of 20 eyes (40%) following AMT, although only six eyes (30%) met their definition of failure. Two recurrences were of a lower stage and did not cause functional disability. All four eyes with preoperative severe aqueous deficiency failed, and four of the six failed eyes had undergone previous conjunctival surgery [7].

Tseng et al. paired AMT with intraoperative MMC (0.04% for five minutes to the deep fornix) in 18 eyes with severe cicatricial disease, most of which had already failed reconstruction, including eyes with previously failed mucous membrane grafting. A deeper, non-inflamed fornix with a continuous tear meniscus was achieved in all eyes, and conjunctival inflammation fell from grade III preoperative to grade 0 in every eye. However, because their definition of success also required freedom from motility restriction, three eyes with focal recurrent adhesion and partial restriction of motility were classified as partial successes, giving a complete success rate of 15 of 18 (83.3%) [6]. The efficacy of AMT depends on severity. In the largest comparative series, lysis and AMT alone achieved overall success in nearly all grade I and II eyes. Complete success was achieved in only three of seven grade 3-4 eyes, with failure in two, as the conjunctival deficit was too large for a membrane to bridge (Table 1) [3,5].

Amniotic membrane alone has also been studied in advanced autoimmune disease. Barabino et al. performed AMT in nine eyes with late-stage ocular cicatricial pemphigoid (OCP) and measured inferior fornix depth serially. They found that fornix depth improved substantially after AMT but gradually declined over 28 weeks, with recurrent symblepharon developing in four eyes (44.4%) [10]. These findings support AMT as a useful component of staged reconstruction but also suggest that its durability may be limited in severe, progressive cicatrizing disease. Overall, AMT is best suggested for lower-grade disease with adequate residual conjunctiva, whereas cases of more extensive tissue loss or persistent inflammation may require additional grafting and sustained control of the underlying disease.

A more recent retrospective cohort evaluated longer-term outcomes in eyes with ocular MMP or drug-related secondary pemphigoid undergoing fornix reconstruction with AMT, buccal mucosal grafting, or Wies cicatricial entropion repair. Reformation of symblepharon with loss of fornix depth occurred in all MMP eyes at a mean of 6.4 ± 7.0 months postoperatively and in 75.0% of eyes with secondary pemphigoid. Trichiasis recurred in all MMP patients by the final visit. Despite these recurrences, both groups reported short-term symptomatic improvement [13]. The cohort was small and retrospective and combined multiple surgical procedures, so the reported recurrence rates cannot be attributed to any specific technique. Nevertheless, these findings are consistent with earlier evidence that anatomical gains in progressive autoimmune disease may not be durable.

Recently, cryopreserved ultra-thick umbilical cord graft - a direct anatomic continuation of the amniotic membrane - has been used for conjunctival surface and fornix reconstruction. Compared with conventional amniotic membrane, umbilical cord graft offers higher tensile strength, longer-lasting epithelial barrier function, and easier surgical handling. Slentz et al. reconstructed the superior or inferior fornix in three patients with anophthalmic socket contracture using a 3.0 x 4.0 cm sheet sutured to the conjunctival defect; the vertical dimension was left untrimmed to provide forniceal depth. The graft was secured to the orbital periosteum using three bolsters passed through a silicone retinal band. The graft handled well without tears or folds, and there was no occurrence of infection, wound dehiscence, or recurrent contracture. Prosthesis fit was judged to be excellent by an independent ocularist in all patients at 10-14 months [19]. Nonetheless, the series was small, involved anophthalmic sockets rather than ocular surface cicatrization, and did not report on fornix depth. Further comparative studies will be helpful to determine whether these properties of ultra-thick umbilical cord graft translate into improved outcomes in fornix reconstruction.

(II) Oral mucosa graft (OMG): It is particularly useful for grade III-IV disease, where amniotic membrane alone may be insufficient to compensate for the extensive loss of conjunctival volume. In the severity-stratified analysis by Kheirkhah et al., adding oral mucosa or conjunctival autograft to AMT raised overall success to 11 of 11 eyes in the grade III-IV subset, compared with five of seven eyes using lysis, AMT, and anchoring sutures alone [3]. Unlike a membrane, the OMG physically replaces missing fornix lining with autologous tissue. Oral mucosa is readily accessible, abundant, and lined by a non-keratinized epithelium that is histologically similar to conjunctiva.

In a prospective study, Kheirkhah et al. combined cicatrix lysis, intraoperative MMC, oral mucosal transplantation, and sutureless AMT in 32 eyes with grade III-IV symblepharon. They achieved complete anatomical and functional success in 84.4%, partial success in 9.4%, and failure in 6.2% [5]. Complete success was higher in grade III than grade IV disease (89.5% vs 76.9%), suggesting that outcomes decline as the extent of cicatrization increases. Preoperative motility restriction was present in 28 of 32 eyes (87.5%). All eyes maintained free ocular movement throughout follow-up, suggesting that layered reconstruction can deepen a severely contracted fornix without compromising motility [5]. However, most eyes were affected by chemical or thermal burns, and patients with severe dry eye despite punctal occlusion were excluded, hence limiting generalizability to eyes with severe aqueous deficiency and other etiologies.

Longer follow-up is available from a small series in which a full-thickness oral mucosa autograft was sutured directly to bare sclera 1 to 3 mm behind the limbus. The amniotic membrane was placed over the rectus muscles, and adjacent bare sclera and the fornix were secured with deepening bolster sutures beneath a temporary tarsorrhaphy and a symblepharon ring. At three years, all seven eyes showed graft integration without recurrent symblepharon. Two eyes developed mild superior entropion, and two developed mild strabismus [20]. The series is small and non-comparative, and no success criterion was prespecified, but it is one of only two included studies reporting a follow-up of three years or more.

As a novel technique that has gained popularity over the past decade, fornix reconstruction using a labial mucous membrane graft combined with minor salivary gland transplantation has shown favorable outcomes in patients with Stevens-Johnson syndrome [21]. This approach does not substantially enlarge the donor site wound while allowing fuller utilization of the harvested tissue, since minor salivary glands are typically trimmed away in conventional labial mucosal graft harvesting to maintain a thin graft profile. In a prospective non-comparative series of 19 patients with severe symblepharon and Schirmer I values of 0, split-thickness labial mucous membrane grafts were used for symblepharon release, while the attached minor salivary glands were positioned in the superior and inferior fornices. At six months, Schirmer I values had improved in 14 eyes (73.7%), corneal transparency improved in 72.2% of eyes, and tear production was greater in eyes receiving more than 10 glands. All grafts remained intact, although six patients (31.6%) required additional surgery for residual symblepharon or cicatricial entropion [21]. Because the study focused primarily on dry-eye outcomes and did not provide detailed fornix depth data, the evidence of labial mucous membrane grafts for anatomic fornix reconstruction is limited.

Reported complications after combined oral mucosa and amniotic membrane surgery include pyogenic granuloma in four of 32 eyes (12.5%), entropion in two eyes (6.3%), and ocular surface keratinization in one eye. Notably, the single case of keratinization occurred in a patient with graft-versus-host disease and was attributed by the authors to an increased evaporative surface rather than to intrinsic keratinization of the graft. No significant donor-site complications were reported in this series [5]. Reported morbidity from oral mucosal harvest in other surgical fields includes contracture, persistent discomfort, and neurosensory deficits. These data primarily derive from urological reconstruction where substantially large grafts are harvested and therefore are an extrapolation rather than direct ophthalmic evidence [22]. Overall, OMG is best supported as a tissue-replacement strategy for advanced grade III-IV disease, particularly when residual conjunctiva is insufficient to restore the fornix after cicatrix lysis.

(III) Conjunctival autograft: Conjunctival autograft from the contralateral eye is histologically the most appropriate lining for fornix reconstruction. It provides the same non-keratinized epithelium, goblet cells, and basement membrane that the fornix has lost, unlike oral or nasal mucosal grafts which provide a different epithelial phenotype. Furthermore, the conjunctival autograft restores the functions of the recess (i.e., tear reservoir, mucin secretion) rather than simply covering it [2,18]. Its use is limited by the availability of healthy donor conjunctiva, and it is often unavailable in bilateral cicatrizing disease such as pemphigoid or Stevens-Johnson syndrome [2].

Data regarding direct outcomes are limited. In the largest series, Kheirkhah et al. used conjunctival autograft in only four of 61 eyes, all with grade III-IV disease. Overall success was achieved in all four but complete success in three, with one partial success requiring reoperation [3]. Given the very small sample, these findings are insufficient to establish superiority over oral mucosa grafting. Thus, conjunctival autograft may be particularly useful when healthy donor conjunctiva is available, but its role is constrained by donor-tissue availability and the limited outcome data.

(IV) Alloplastic materials: They have been described as a temporary barrier in situations where biological grafting is undesirable or unavailable, but the evidence for their use in fornix reconstruction is limited. Levin et al. applied a non-porous polytetrafluoroethylene (polytef) membrane in nine patients, which was removed after two to three weeks once residual conjunctival epithelium had grown beneath it. Satisfactory results were reported in eight of nine patients, although one developed recurrent contraction at four months. The series was heterogeneous, comprising OCP, tumor resection, radiation-related scleral melt, and contracted sockets, and the outcome measure was not defined [23].

A more durable approach anchors the alloplastic material to the orbital rim to resist contractile forces. Demirci et al. reconstructed fornices in 17 patients using an expanded polytetrafluoroethylene (e-PTFE) sheet draped over a rigid 0.8-mm nylon foil strip anchored to the orbital rim. The composite stent was removed after a mean of five weeks. After a mean follow-up of 47 months, prosthesis retention improved in all patients and was considered entirely satisfactory in 15 (88%). The reconstructed fornix was described as deep in seven patients (41%) and adequate in nine (53%). Complications included localized lower-eyelid infection in two patients (12%) and pyogenic granuloma in three (18%). Four patients (24%) with extensive involvement required staged reconstruction [24]. Because this series involved exclusively anophthalmic sockets, its application to ocular surface disease in the seeing eye is an extrapolation. More recent data are available for e-PTFE applied at the ocular surface rather than in the socket. A retrospective series analyzed 23 eyes of 22 patients undergoing symblepharon lysis with application of a polytetrafluoroethylene sheet over the sclera following chemical injury, with a minimum of 12 months of follow-up. Success was reported in 52.2% and partial success in 34.8%, with the remainder classified as failures. Recurrence occurred at a mean of 6.75 ± 3.6 months, and failures were concentrated in younger patients (mean age: 9.75 years) and in grade IIIc disease. No sight-threatening complications were reported [25]. The outcome definitions were specific to that series and are not directly comparable with those used in the biological grafting literature. Taken together, alloplastic materials are best regarded as a temporary barrier or salvage option where biological grafting has failed, or donor tissue is unavailable, rather than as an established alternative to biological grafting.

(V) Cultivated epithelial transplantation: Cultivated epithelial techniques expand a small biopsy of the patient's own epithelial cells into a transplantable sheet, restoring the deficient ocular surface with autologous tissue while requiring only a minimal donor sample [26]. In severe cicatricial ocular surface disease, cultivated epithelial transplantation is generally one part of a staged reconstructive strategy. Successful transplantation depends on a stable ocular surface, including adequate fornix depth and control of inflammation [6].

The dependence of subsequent reconstruction on an adequate fornix is well illustrated by the series of Tseng et al., in which restoration of a deep fornix and an adequate tear reservoir preceded successful keratolimbal allograft in nine of 18 eyes, six of which went on to penetrating keratoplasty [6]. These findings underscore the role of fornix reconstruction as a foundational step in staged ocular surface rehabilitation.

Cultivated oral mucosal epithelial transplantation (COMET) is the cultivated technique most directly applicable to fornix reconstruction. A small oral mucosal biopsy is expanded in culture on a denuded amniotic membrane for about two weeks, and the resulting epithelial sheet is transplanted to reconstruct the ocular surface and fornix [27]. Because only a small biopsy is required (2 mm² in one series), COMET may reduce donor-site morbidity compared with harvesting a full-thickness OMG [9]. Clinical evidence, while limited, suggests that COMET can provide durable reconstruction in selected severe cases. Komai et al. demonstrated long-term outcomes of COMET for fornix reconstruction in eyes with chronic cicatrizing disease. They reported a five-year success probability of 79.6% ± 10.7% across 16 eyes. Outcomes appeared to depend on the etiology; success probability was 100% for thermal or chemical injury and 53.3% ± 24.8% for OCP, but this difference did not reach statistical significance (p = 0.53), and the subgroup estimates were based on very small numbers [8].

In a separate series of 19 eyes with grade III-IV symblepharon (10 chemical injury, eight Stevens-Johnson syndrome, and one OCP), Gopakumar et al. performed COMET for fornix reconstruction. They achieved a deep fornix with unrestricted motility in 11 eyes (57%) and partial reconstruction in five eyes (26%), with failure in three eyes (16%) [9]. Successful fornix formation allowed for subsequent visual rehabilitation in six eyes, whereas some partial successes required a type 2 rather than type 1 keratoprosthesis because the reconstructed fornix remained inadequate [9]. Overall, COMET appears to be a promising option for severe cicatrizing disease when it is important to minimize donor-site tissue requirements. The current evidence is limited to small, non-comparative series, and there are no direct data demonstrating superiority over oral mucosal or conjunctival grafting.

Allogeneic cultivated limbal epithelial sheets have also been used for symblepharon release and fornix reconstruction after severe ocular burns. In the largest series included in this review, 80 eyes graded with the severity system described above achieved complete success in 40 (50.0%), partial success in 25 (31.3%), and failure in 15 (18.8%) after the first procedure. Of the 27 eyes that underwent a second procedure, 21 (77.8%) achieved complete success. Complete success was 63.0% in eyes with mild or no preoperative inflammation compared with 23.1% in eyes with moderate or severe inflammation (p < 0.001, chi-square). Corneal surface conditions improved in 43 eyes (53.8%), allowing subsequent keratoplasty in 22 [11]. Because the epithelial sheets were allogeneic, this approach carries a risk of immune rejection that autologous techniques do not. Rejection occurred in seven eyes (8.8%) and was controlled medically. The series was retrospective and non-comparative, and its definition of partial success was score-based rather than anatomical, which limits direct comparison with the mucosal grafting literature.

Table 2 summarizes the individual studies supporting this section, including study design, sample size, etiology, follow-up duration, and definition of success. This allows for the strengths and limitations of the available evidence to be appropriately assessed (Table 2).

**Table 2:** Primary clinical studies of fornix reconstruction in ocular surface disease This table includes studies reporting fornix outcomes after surgical reconstruction for cicatricial ocular surface disease. Six additional included studies are discussed in the text but are excluded here because they do not report comparable outcomes. Abbreviations: AMT, amniotic membrane transplantation; COMET, cultivated oral mucosal epithelial transplantation; MMP, mucous membrane pemphigoid; MMC, mitomycin C; OCP, ocular cicatricial pemphigoid; OMG, oral mucosa graft; PTFE, polytetrafluoroethylene; SJS, Stevens-Johnson syndrome; TEN, toxic epidermal necrolysis

Study/Design	Eyes/Patients	Etiology and severity	Intervention	Follow-up	Definition of success	Key outcomes
Levin & Dutton, 1990 [23], Retrospective case series	9 patients	Mixed indications, including OCP, tumor resection, post-radiation disease, contracted socket	Temporary PTFE membrane placed over reconstructed surface and removed after epithelialization	Up to 18-21 months	Not formally defined; “satisfactory result”	8/9 patients had satisfactory result. One developed recurrent contraction at 4 months. Historical study with heterogeneous indications.
Solomon et al., 2003 [2], Noncomparative interventional case series	17 eyes/15 patients	Mixed: OCP, SJS/TEN, allergic conjunctivitis, prior surgery, trauma	Cicatrix lysis, conjunctival recession, and AMT with lid-anchoring sutures	Mean 37 months	Deep fornix without recurrent scar or motility restriction at ≥6 months; partial success allowed focal recurrence without functional restriction	Complete success in 12/17 (70.6%); partial success in 2/17; failure in 3/17. Outcomes were poorer in autoimmune disease and previously operated eyes.
Barabino et al., 2003 [10], Prospective noncomparative case series	9 eyes/9 patients	Advanced OCP	Excision of inflamed/fibrotic tissue and AMT with fornix-anchoring sutures	Mean 48 weeks	Serial fornix depth, symblepharon recurrence, goblet-cell recovery, and inflammation	No symblepharon through 16 weeks; 4/9 (44%) developed recurrence by 28 weeks. Fornix depth improved substantially.
Jain & Rastogi, 2004 [7], Prospective noncomparative study	20 eyes/18 patients	Recurrent pterygium, chemical/thermal burns, SJS, congenital disease	Symblepharon excision and AMT; fornix-forming silk sutures used in selected eyes	Mean 53 weeks	Complete epithelialization without recurrence, or limited recurrence without functional or cosmetic disability	6/20 (30%) failed by study criteria and 8 / 20 (40%) had recurrence. All 4 eyes with Schirmer <5 mm failed.
Tseng et al., 2005 [6], Noncomparative interventional case series	18 eyes/16 patients	Chemical / thermal burns, recurrent pterygia, SJS/TEN, OCP	Cicatrix lysis, intraoperative 0.04% MMC, AMT, and lid-anchoring sutures	Mean 14 months	Stable deep fornix without scar, inflammation, or motility restriction at ≥6 months	Success in 15/18 (83.3%); partial success in 3/18. Fornix depth improved, and inflammation decreased in all eyes.
Kheirkhah et al., 2008 [3], Retrospective comparative case series	61 eyes/47 patients	SJS, chemical burns, chronic cicatricial conjunctivitis, pterygium, MMP, other causes	Cicatrix lysis and AMT with sutures or fibrin glue, with or without 0.04% MMC; selected eyes received anchoring sutures and mucosal or conjunctival grafts	Mean 25 months	Complete success: deep fornix without scar or motility restriction. Partial success: focal recurrence. Failure: recurrent symblepharon	Overall success of 85.2% after initial surgery and 96.7% after reoperation. AMT alone was highly successful in Grade I-II disease; adding mucosal/conjunctival grafts achieved success in 11/11 eyes with Grade III-IV disease.
Kheirkhah et al., 2013 [5], Prospective noncomparative case series	32 eyes/32 patients	Grade III-IV disease from chemical / thermal burns, SJS, MMP, other causes	Cicatrix lysis, 0.04% MMC, OMG, sutureless AMT with fibrin glue, and anchoring sutures	Mean 16 months	Deep fornix without scar or motility restriction; partial success allowed focal recurrence	Complete success in 27/32 eyes (84.4%), partial success in 3/32, failure in 2/32. No postoperative motility restriction occurred in eyes with preoperative restriction.
Gopakumar et al., 2019 [9], Retrospective noncomparative case series	19 eyes/19 patients	Grade III-IV symblepharon from chemical injury, SJS, OCP	Symblepharon release ± 0.04% MMC, COMET, AMT, anchoring sutures, and large-diameter bandage contact lens	Mean 18 months	Success: deep fornix without restriction. Partial success: focal recurrence. Failure: recurrent symblepharon	Success in 11/19 eyes (57%), partial success in 5/19, failure in 3/19. Failures occurred in severe chemical injuries and one SJS eye with dry eye.
Komai et al., 2022 [8], Prospective interventional case series	16 eyes	OCP, chemical / thermal injury, other chronic cicatrizing diseases	Symblepharon release with COMET	Up to 5 years	Success probability based on standardized ocular-surface scores for symblepharon and fornix shortening	Five-year success probability of 79.6% ± 10.7%. Outcomes appeared better after chemical / thermal injury than OCP, although the difference was not statistically significant (p = 0.53).
Sant’Anna et al., 2012 [21], Prospective noncomparative interventional case series	19 eyes/19 patients	Severe symblepharon and dry eye secondary to SJS; Schirmer I = 0 in all eyes	Split-thickness labial mucous membrane graft for symblepharon release with transplantation of attached minor salivary glands into the superior and inferior fornices	6 months	No single endpoint defined; outcomes focused on tear production, corneal transparency, and visual acuity	Schirmer, I improved in 14/19 eyes (73.7%); corneal transparency improved in 72.2% of eyes; visual acuity improved in 8/19 (42.1%). All grafts remained intact. 6 patients required repeat surgery for residual symblepharon and entropion.
Zhao et al., 2015 [28], Retrospective interventional case series	16 eyes/4 patients	SJS, chemical and thermal burns, OCP, post-tumor excision, and immune dysregulation; 12 eyes had prior surgery	Conjunctival recession with closure of the gap between recessed conjunctiva and Tenon’s capsule using a running 9-0 nylon suture + AMT; OMG added when conjunctiva was insufficient. No MMC or anchoring sutures	Mean 17 months	Complete success: deep fornix, quiet ocular surface, and full motility. Partial success: incomplete restoration	Complete success in 13/16 eyes (81.3%), partial success in 2/16, failure in 1/16. Motility and inflammation improved significantly.
Cheng et al., 2017 [11], Retrospective interventional case series	80 eyes/80 patients	Chemical and thermal burns; symblepharon graded I-IV using the same severity system applied elsewhere in this review	Symblepharon lysis with allogeneic cultivated limbal epithelial sheet carried on human amniotic membrane; bandage contact lens; systemic and topical corticosteroids with topical cyclosporine	Mean 26.4 months	Complete success: anatomically deep fornix without scar or motility restriction. Partial success: recurrence score more than 4 points below preoperative score. Failure: less than 4 points below	Complete success in 40/80 eyes (50.0%), partial success in 25/80 (31.3%), failure in 15/80 (18.8%). Complete success was 85.0% in grade I/II, 51.5% in grade III, and 22.2% in grade IV (p = 0.001). Complete success of 63.0% with mild or no preoperative inflammation vs 23.1% with moderate or severe (p < 0.001). 21/27 (77.8%) had complete success after a second procedure. Immune rejection reported in 7 eyes (8.8%).
Martinez-Osorio et al., 2021 [20], Retrospective interventional case series	7 eyes/7 patients	Severe symblepharon after chemical or fireworks burn	Oral mucosa autograft (mean 3 x 2 cm) sutured to bare sclera 1-3 mm behind the limbus, with AMT over the rectus muscles and adjacent sclera. Fornix-deepening bolster sutures, temporary tarsorrhaphy, and symblepharon ring over a bandage contact lens	3 years	Not formally defined; graft integration and absence of recurrent symblepharon	All grafts integrated with no recurrence of symblepharon at 3 years. Mild superior entropion in 2 patients and mild strabismus in 2.
Venugopal et al., 2024 [25], Retrospective interventional case series	23 eyes/22 patients	Chemical injury; Grade 3 length in 10 eyes and Grade 3 width in 12 eyes	Symblepharon lysis with expanded polytetrafluoroethylene sheet applied over the sclera	Minimum 12 months	Success, partial success, and failure defined within the study; criteria not aligned with the anatomical definitions used in the other series	Success in 52.2% and partial success in 34.8%, with the remainder classified as failures. Mean time to recurrence of 6.75 ± 3.6 months. Failures concentrated in younger patients (mean age 9.75 years) and Grade 3c disease. No sight-threatening complications.
Khan et al., 2024 [13], Retrospective cohort	14 eyes/12 patients	Ocular mucous membrane pemphigoid and drug-related secondary pemphigoid	Fornix reconstruction with amniotic membrane and/or buccal mucosal graft (8 eyes) or Wies cicatricial entropion repair (7 eyes); 2 eyes received both	Mean 22.7 months (MMP); mean 15.4 months (secondary MMP)	Maintenance of fornix depth at latest follow-up	No MMP eye retained fornix depth at final follow-up; recurrence documented in 7/10 eyes at a mean of 6.4 ± 7.0 months. Trichiasis recurred in all MMP patients. 3/10 MMP eyes required further surgery. In secondary MMP, 3/4 eyes (75.0%) lost fornix depth and 2/3 eyes with preoperative trichiasis re-formed it. Short-term symptomatic improvement in both groups.

Surgical Techniques for Fornix Reconstruction

No study has compared surgical techniques while holding graft material constant. Because the two vary together across the available series, their individual contributions to outcomes cannot be disentangled. Nevertheless, the published series consistently emphasize the same core technical steps: complete release of adhesions, secure fixation of the graft at the fornix depth, and stabilization of the reconstruction until healing is complete. Incomplete execution of these steps is the most common explanation proposed for early recurrence, although this attribution is retrospective and has not been directly tested [3].

The fundamental first step is cicatrix lysis and conjunctival recession, which involves completely releasing the subconjunctival scar and the adhesions binding the palpebral to the bulbar conjunctiva. The freed conjunctival flap is then recessed into the depth of the fornix, and the exposed episclera is resurfaced with graft material [3]. Complete scar removal appears important for preventing early re-tethering. Tseng et al. attributed previous surgical failures to persistent inflammation from incompletely excised subconjunctival fibrovascular tissue extending toward the limbus [6]. Dissection should continue toward the fornix until healthy Tenon's capsule is encountered. In a series of 16 eyes with mixed-etiology symblepharon, closure of the gap between recessed conjunctiva and the cut edge of Tenon's capsule with a running 9-0 nylon suture resulted in complete success in 13 eyes (81.3%) and failure in one eye (6.3%) [28].

During dissection, orbital fat may occasionally be visualized. It has not been studied whether exposed orbital fat influences re-adhesion in the fornix. In upper eyelid surgery, preaponeurotic fat advancement flaps have been used to prevent unintended re-adhesion [29]. Extrapolation of that observation to forniceal reconstruction is speculative and is not supported by any fornix-specific data. Kheirkhah et al. similarly excised scar tissue around the rectus muscle insertions to restore full globe motility before graft placement [5]. AMT over the exposed sclera may further reduce re-adhesion by providing a barrier between the recessed conjunctiva and subconjunctival tissue [2,18].

Anchoring sutures can then be used to secure the recessed tissue and graft at the depth of the new fornix, holding the reconstruction against the contractile forces of healing. The sutures are passed full-thickness through the eyelid and tied over external bolsters to keep the suture from cutting through the skin [2]. Silicone retinal band has also been described as bolster material for fornix-deepening sutures, with three bolsters passed through the band to secure a graft to the orbital periosteum [19]. Comparative data on bolster material are not available. Kheirkhah et al. found that additional anchoring sutures achieved overall success in 100% of grade I, 70% of grade II, and 71.4% of grade III-IV eyes [3]. In their multivariate analysis, anchoring sutures showed a seemingly negative correlation with complete success (p = 0.006), but they were also preferentially used in eyes with canthal involvement and other markers of difficult disease. The authors interpreted this as confounding by indication rather than a harmful effect of the sutures. The use of anchoring sutures has produced favorable outcomes, particularly in severe symblepharon combined with mucosal grafting [5]. Anchoring sutures should therefore be considered selectively rather than routinely, and the negative statistical association should not be interpreted as a reason to avoid them in severe disease.

Fibrin glue has become a useful alternative to sutures for securing amniotic membrane in the fornix. It shortens operative time, reduces postoperative inflammation, and improves comfort by eliminating the foreign-body sensation associated with sutures [30]. This evidence comes primarily from pterygium surgery and is extrapolated to fornix reconstruction. Harley et al. used fibrin glue with small curved hooks to position the amniotic membrane within the fornix and canthus after excision of conjunctival tumors, reporting good outcomes in a small series [31]. In the series by Kheirkhah et al., fibrin glue was used in 27 of 61 eyes, and the fixation method was not significantly associated with surgical outcome on multivariate analysis [3]. A subsequent prospective series used sutureless fixation with fibrin glue in all cases [5].

Symblepharon rings and conformers may be used postoperatively to help maintain fornix depth through the weeks of wound remodeling, when contractile forces are greatest [32]. Their use has been inconsistently reported and has not been formally compared with no retaining device. Some studies used symblepharon rings routinely, while others used large-diameter bandage contact lenses [5,9,32]. Their role is hence best considered as supportive and dependent on technique.

Medical Management of Fornix Reconstruction

Postoperative inflammation is consistently identified in these series as the setting in which recurrent scarring develops. Control of inflammation is therefore a central component of postoperative care, although no study has tested whether more intensive anti-inflammatory treatment reduces recurrence. The reported intensity of immunomodulation varies with etiology, from an intraoperative antifibrotic alone after a single insult burn to long-term systemic immunosuppression in autoimmune disease [33]. This section is organized accordingly; antifibrotic agents used at the time of surgery, topical anti-inflammatory therapy in the postoperative period, and systemic immunosuppression are considered separately for each underlying disease.

(I) Antifibrotic agents: Antifibrotic agents can be used intraoperatively to limit scarring at the time of reconstruction, and postoperatively as serial injections in progressive cicatrization. Intraoperative mitomycin C (MMC) is a useful antifibrotic adjunct in selected cases. MMC is an alkylating agent that inhibits fibroblast proliferation and collagen deposition, and it is applied to the deep fornix to limit scarring before the graft is placed [34]. In the fornix reconstruction literature, the reported regimens are relatively consistent: 0.04% MMC applied to the deep fornix for approximately three to five minutes, with some authors adjusting its use according to the severity of scarring or inflammation [3,5,6,9]. In each case, the sponges were placed beneath the recessed conjunctiva while avoiding direct contact with bare sclera, followed by removal of the sponges and copious irrigation [3,6].

Evidence supporting MMC in fornix reconstruction is primarily observational, and the principal series is described above [6]. In the larger series by Kheirkhah et al., MMC was used in 47 of 61 eyes and was positively associated with complete success on multivariate analysis (p = 0.001) [3]. However, this association does not establish causation, as the treatment was not randomized and MMC may have been selected based on clinical characteristics.

Higher concentrations and longer exposure to MMC increase the risk of complications such as delayed epithelialization, scleral thinning, tissue necrosis, and other forms of ocular surface toxicity [35]. No MMC-related complication was reported in any of the fornix series reviewed in this study, where application was limited to the subconjunctival fornix rather than the bare sclera [3,5,6]. Nevertheless, MMC is best reserved for eyes at higher risk of recurrent fibrosis, where the benefit of suppressing scar formation appears to outweigh the risk of MMC-associated toxicity. Long-term surveillance remains appropriate as late scleral complications can occur [35].

5-Fluorouracil (5-FU) is an alternative antimetabolite that inhibits thymidylate synthase, thereby reducing fibroblast proliferation and collagen synthesis. It appears to be less potent than MMC; in vitro, a five-minute exposure to mitomycin at 1,000 μg/mL was cytocidal to human Tenon's capsule fibroblasts, while 5-FU remained cytostatic even at 25,000 μg/mL [36]. Clinical evidence for 5-FU in cicatrizing conjunctival disease is limited to serial injections rather than intraoperative application. In a retrospective cohort of 112 eyes with OCP or SJS/TEN, 25 eyes received one or more injections of 0.5-1.0 mL of 50 mg/mL in the scarred conjunctiva at three- to five-week intervals. 5-FU treatment was associated with improved final visual acuity, less corneal scarring and less severe symblepharon, and fewer mucous membrane graft procedures. The reduction in symblepharon was most apparent in eyes receiving five or more injections [14]. However, the study did not specifically report fornix-depth outcomes. No studies have evaluated 5-FU as an intraoperative adjunct to fornix reconstruction. Its potential intraoperative use is extrapolated from filtration surgery, where a systematic review found a small reduction in surgical failure at one year but an increased risk of complications [37].

(II) Topical anti-inflammatory therapy: Topical anti-inflammatory therapy extends the control of fibrosis into the postoperative period, during which the remodeling fornix is most vulnerable to recurrent scarring. Corticosteroid drops are first-line and are typically given in a tapering regimen over several weeks [38]. Reported postoperative regimens vary; some use topical antibiotics until epithelialization is complete, with steroids tapered according to conjunctival inflammation, whereas more severe grade III-IV disease may require antibiotics for approximately three weeks and steroids for up to three months [3,5]. Preservative-free drops are preferred to avoid adding preservative toxicity to the already compromised ocular surface [39].

Persistent postoperative inflammation around the surgical site should be anticipated, particularly in severe disease. In the series reporting this complication, it was resolved with subconjunctival triamcinolone acetonide (Table 3) [3,5]. Topical calcineurin inhibitors (e.g., cyclosporine, tacrolimus) serve as steroid-sparing agents when prolonged anti-inflammatory therapy is needed or when steroid-related complications (e.g., glaucoma, cataract) develop [40].

**Table 3:** Complications and causes of failure in fornix reconstruction of the ocular surface Abbreviations: AMT, amniotic membrane transplantation; IOP, intraocular pressure; MMC, mitomycin C; OMG, oral mucosa graft

Complication	Reported frequency	Contributing factors	Prevention and management
Recurrent symblepharon and fornix re-shortening	Failure after first surgery: 9/61 (14.8%), decreasing to 2/61 after reoperation [3]. Failure: 2/32 (6.2%) in grade III-IV disease [5]. Recurrence ranged from 17-44% in other series [2,9,10].	Incomplete scar removal; residual fibrovascular tissue; severe symblepharon; canthal involvement; persistent inflammation; active autoimmune disease [3,5,6,10].	Complete cicatrix lysis; adequate graft coverage; secure fornix fixation; selective MMC in high-risk eyes; aggressive postoperative inflammation control; early follow-up [3,5,9].
New symblepharon at the surgical margin	3/61 (4.9%) developed new symblepharon at the edge of the surgical field within 2-4 months [3]. One additional case occurred in an eye with grade IV disease [5].	Surgical trauma to adjacent conjunctiva; progressive underlying disease; tissue fragility [3,5].	Extend lysis and resurfacing beyond the visibly involved area when possible; closely monitor the surgical margins [3,5].
Graft shrinkage, focal scarring, or cicatricial entropion	Entropion: 2/32 (6.3%) [5]. Focal recurrent scarring occurred in 9-26% across several studies [2,3,5,9].	Graft contraction during healing; inadequate graft size; deep fornix scarring [5].	Harvest oral mucosa graft approximately 30% larger than defect to account for contraction; thin the graft, secure it deeply in the fornix; treat inflammation early [5].
Ocular surface keratinization	1/32 (3.1%) after oral mucosa + AMT; 1/17 (5.9%) after AMT alone [2,5].	Severe underlying dry eye and increased evaporative surface, not necessarily metaplasia of the graft itself [5].	Optimize tear function before surgery; defer reconstruction in severe untreated aqueous deficiency; use lubrication and punctal occlusion/cautery when appropriate [5,7,9].
Pyogenic granuloma	7/61 (11.5%), 4/32 (12.5%), and 2/17 (11.8%) in reported series, usually occurring within 1-4 months [2,3,5].	Postoperative inflammation at graft or suture sites; exposed suture material [2,3,5].	Subconjunctival triamcinolone; surgical excision for large or persistent lesion [2,3,5].
Persistent postoperative inflammation	5/61 (8.2%), 5/32 (15.6%), and 4/20 (20%) in different series [3,5,7].	Underlying disease activity; extensive surgery; suture material; incomplete removal of inflamed tissue [3,5,7].	Taper topical steroids according to inflammation and use preservative-free drops; consider subconjunctival triamcinolone for focal severe inflammation and calcineurin inhibitors when long-term steroid-sparing therapy is needed [3,5,39,40].
Donor-site morbidity (oral mucosa)	No significant donor-site complications were reported in 32 consecutive patients undergoing OMG [5]. Contracture, persistent discomfort, and neurosensory deficit are described in urological reconstruction literature [22].	Graft size and thickness; selection of donor site [22].	Harvest a thin graft with minimal submucosa; allow additional tissue for postoperative contraction; consider cultivated epithelial techniques when a large graft is required [5,9].
MMC-associated toxicity	No MMC-associated complications were reported in the fornix reconstruction literature reviewed [3,5,6]. Other ophthalmic studies reported delayed epithelialization, scleral thinning, necrosis, and late scleral complications [35].	Higher concentration or prolonged exposure; direct application to bare sclera; ischemic recipient bed [6,35].	Use 0.04% MMC for 3-5 minutes in the deep fornix beneath recessed conjunctiva; avoid direct contact with bare sclera; reserve MMC for eyes at high risk of recurrent fibrosis and monitor for late complications [3,6,35].
Suture, conformer, and retaining-device complications	Not reported in the fornix literature. Suture-related skin injury and foreign-body symptoms have been described [2,30]. Alloplastic fornix stenting was associated with localized eyelid infection in 2/17 patients and pyogenic granuloma in 3/17 [24].	Tension from full-thickness sutures; prolonged device retention; poor device fit [2,9,32].	Use external bolsters with full-thickness sutures; remove bolsters within 1-2 weeks; select appropriately sized symblepharon rings or conformers [3,5,32]
Corticosteroid-related complications	Increased IOP occurred in 1/32 patients after subconjunctival triamcinolone [5].	Prolonged or repeated corticosteroid exposure; steroid responsiveness [5,40].	Monitor IOP during prolonged steroid therapy; consider topical calcineurin inhibitors as steroid-sparing therapy in case of prolonged treatment [40].
Failure related to tear film insufficiency	All 4 eyes with Schirmer <5 mm failed in one series [7]. Severe dry eye was excluded from two later series [5,9].	Severe aqueous deficiency; loss of goblet cells; keratinized recipient surface [2,7].	Assess and optimize tear function before surgery; consider punctal occlusion or cautery when appropriate. In severe tear deficiency, consider delaying reconstruction or using epithelial graft [7,9].

(III) Systemic immunosuppression by underlying disease: The need for systemic therapy and the timing of reconstruction primarily depend on the underlying cause of cicatrization. Mucous membrane pemphigoid (MMP) requires control of ongoing systemic inflammation, while SJS/TEN and stable traumatic or chemical disease generally do not. These conditions are therefore considered separately.

(III.A) Mucous membrane pemphigoid (including OCP): Ocular MMP is a chronic, progressive cicatrizing conjunctivitis in which ongoing inflammation continues to cause fibrosis even after surgery [12,41]. Systemic immunosuppression is therefore a central component of management, particularly in severe or progressive disease. Consensus recommendations include prednisone with cyclophosphamide for severe disease, with agents such as azathioprine or dapsone for less severe cases. Intravenous immunoglobulin may be considered for treatment-resistant disease [12].

The largest outcome series by Saw et al., involving 223 eyes of 115 patients with ocular MMP, supports a stepwise approach to systemic therapy. They defined success as induction of quiescence with a quiet, white eye sustained for at least three months. Across 388 treatment episodes, 50% were classified as successes, 27% as qualified successes, and 23% as failures. Cyclophosphamide had the highest success rate among the major agents (69% success, 21% qualified success, 10% failure), followed by mycophenolate, azathioprine, dapsone, and sulfapyridine. Combination therapy also produced higher response rates than treatments with single agents [42]. However, adverse effects were common and accounted for a substantial proportion of changes in treatment, most frequently with azathioprine and least frequently with mycophenolate [42].

Conjunctival cicatrization progressed in 41% of eyes despite treatment, including some eyes with relatively little active inflammation [42]. Quiescence is defined as the sustained absence of conjunctival inflammation with minimal conjunctival hyperemia, no new symblepharon formation, and no further progression of fornix shortening while on adequate therapy. Quiescence is a necessary condition before elective reconstruction, but it does not necessarily indicate that the underlying cause of cicatrization has stopped. Elective fornix reconstruction should therefore be performed only after the disease has been adequately controlled, with continued postoperative surveillance as recurrence may occur despite a seemingly quiet ocular surface.

B-cell therapy such as rituximab has been reported in refractory disease. Maley et al. retrospectively compared 24 patients receiving rituximab in addition to conventional immunosuppression with 25 patients receiving conventional therapy alone. Disease control was achieved in all 24 patients treated with rituximab, compared with 10 of 25 (40%) on conventional therapy (p < 0.01) [43,44]. However, the study was non-randomized; the treatment groups differed in disease severity, and all patients receiving rituximab had previously failed conventional therapy. Additionally, the outcomes reflect systemic disease control rather than fornix-specific outcomes, and its specific effect on the durability of fornix reconstruction remains uncertain.

(III.B) Stevens-Johnson syndrome and toxic epidermal necrolysis: SJS/TEN differ from MMP in that they are generally an acute, drug-triggered illness rather than a chronically active autoimmune disease. Therefore, the most effective intervention for preventing ocular cicatrization is during the acute phase. Gregory reported a prospective series of 10 consecutive patients in whom amniotic membrane was applied to the lid margins, palpebral conjunctiva, and ocular surface repeatedly while severe inflammation and epithelial sloughing persisted. At six months or more of follow-up, all patients had best-corrected acuity of 20/30 or better, with nine of 10 at 20/20. Dry eye was moderate or less in all patients. Ocular surface and eyelid scarring was mild to moderate, with only mild symblepharon reported in four patients. Notably, no patients received systemic corticosteroids [15]. A subsequent review by Shay et al. reached similar conclusions [16].

Once the acute disease has resolved, chronic SJS/TEN generally does not require systemic immunosuppression for the eye. Definitive fornix reconstruction can be performed once the ocular surface is stable and inflammation has subsided [3,5,9]. Disease management instead focuses on optimizing the ocular surface, particularly the tear film. Severe aqueous deficiency is a significant risk factor for failure of reconstruction [7]. Progressive conjunctival scarring in a patient with previous SJS should nevertheless prompt reassessment, as a small subset of patients may develop a chronic cicatrizing process that resembles MMP [12,42].

(III.C) Stable traumatic and chemical cicatrization: Cicatrization following thermal or chemical burns, mechanical trauma, or repeated surgery generally represents a single insult without an ongoing systemic autoimmune source. Once inflammation has subsided and the ocular surface is quiet, fornix reconstruction can usually proceed without systemic immunosuppression. Topical therapy with an intraoperative antifibrotic treatment is generally sufficient [3,6].

The principal consideration is timing. Kheirkhah et al. operated at least six months after the initial injury in patients with chemical or thermal burns, to allow inflammation to stabilize [5]. A similar interval was applied before COMET in a separate series by Gopakumar et al. [9]. In children, however, a longer interval before surgery may carry a greater risk of amblyopia, and one of the two failures in the prospective series by Kheirkhah et al. was a three-year-old with a severe thermal burn [5].

Outcomes are generally favorable in this group according to the literature. All four post-traumatic eyes achieved complete success in one AMT series by Solomon et al. [2]. Complete success reached 87.5% in the chemical burn and 85.7% in the thermal burn subgroups in the prospective mucosal grafting series by Kheirkhah et al. [5]. Komai et al. reported a five-year success probability of 100% in the thermal and chemical injury subgroup, although it was only three eyes [8]. These results should be interpreted cautiously because the available studies are small. Moreover, a stable etiology does not overcome other risk factors, particularly severe aqueous tear deficiency, which has been associated with reconstruction failure [7].

Complications and Causes of Failure

Fornix reconstruction has a characteristic range of complications, many of which reflect the underlying inflammatory and fibrotic processes responsible for the original disease. Two patterns emerge from the studies reviewed. First, most complications are associated with postoperative inflammation and occur within the first one to four months, which coincides with the period when recurrent symblepharon is most likely to develop [3,5]. This temporal overlap is consistent with a relationship between postoperative inflammatory activity and recurrence. However, co-occurrence does not establish causation by itself, and no study has tested whether more intensive anti-inflammatory treatment reduces recurrence. The indirect evidence is inconsistent. Preoperative inflammatory activity was independently associated with recurrence on multivariate analysis in an 80-eye series, whereas the largest earlier series did not identify inflammatory activity among the factors significantly associated with surgical outcome [3,11]. Second, several complications that were attributed to specific materials (e.g., keratinization with OMG, toxicity from MMC) were uncommon or not observed in the fornix-specific studies. Therefore, these complications should not necessarily be attributed to these materials alone [5,35]. Table 3 summarizes the reported complications, their frequency where available, contributing factors, and strategies for prevention and management.

Discussion

Three findings were consistently observed across the included studies, and their convergence across independent series is more informative than any single study.

The first finding is that the amount of conjunctiva remaining after cicatrix lysis is more consistently associated with surgical results than any other single variable. In one series, lysis and AMT alone achieved overall success in 19 of 20 grade I-II eyes. Complete success was achieved in only three of seven grade III-IV eyes [3]. The same gradient was independently reproduced at a different center using a different graft material. In 80 eyes treated with allogeneic cultivated limbal epithelial sheets, complete success declined from 85.0% in grade I/II to 51.5% in grade III and 22.2% in grade IV disease [11]. This supports the length grade as a measure of the reconstructive problem, specifically the amount of lining that must be replaced, rather than as a property of any particular technique. However, this association does not establish that matching graft material to the grade improves outcomes. No study has randomly assigned graft material within severity grades.

The second finding is that results after a single procedure may underestimate what is ultimately achievable. Overall success increased from 85.2% to 96.7% with additional procedures in one series [3]. In another study, 21 of 27 eyes (77.8%) undergoing a second operation achieved complete success [11]. Severe fornix cicatrization is therefore better viewed as a staged reconstructive process than as a single procedure with a binary outcome. This distinction has implications for both patient counseling and outcome reporting. A series reporting results for a first procedure is not directly comparable with one reporting final anatomical status after repeat intervention.

The third finding is that etiology may influence durability more strongly than the immediate anatomical result. Immediate fornix deepening has been achieved even in advanced autoimmune disease, but this improvement may not be sustained. Fornix depth declined over 28 weeks after AMT in late-stage OCP, with recurrent symblepharon in four of nine eyes [10]. In a more recent cohort, no MMP eye retained fornix depth at the final follow-up, with recurrence documented in seven of 10 eyes at a mean of 6.4 months [13]. In contrast, the five-year success probability after COMET was 100% in the thermal and chemical injury subgroup compared with 53.3% ± 24.8% in OCP [8]. This difference did not reach significance on log-rank testing (p = 0.53). The comparison was also based on only three and five eyes, respectively [8]. The relationship between etiology and outcome should therefore be interpreted cautiously; the direction of effect is consistent across several small subgroups and is biologically plausible, but the largest series did not identify a significant association between etiology and surgical outcome [3].

Two areas of disagreement in the literature also warrant explicit discussion. The first concerns preoperative inflammation. One series did not identify inflammatory activity among the factors that were significantly associated with surgical results [3]. Another study identified preoperative inflammatory activity as an independent predictor of recurrence on multivariate analysis (p = 0.016) [11]. Complete success occurred in 63.0% of eyes with mild or no inflammation, compared with 23.1% of eyes with moderate or severe inflammation (p < 0.001) [11]. The populations also differed, with mixed etiologies at one center and burn injuries at the other. Accordingly, the widely cited inference that preoperative inflammatory quiescence improves outcomes remains based on inconsistent evidence. The second area concerns anchoring sutures. Anchoring sutures were negatively associated with complete success on multivariate analysis in one series, but they were preferentially used in eyes with canthal involvement and other markers of difficult disease [3]. Confounding by indication is therefore a more plausible explanation than a harmful effect of the sutures themselves. The available data cannot exclude other explanations. The association should therefore not be interpreted as evidence that anchoring sutures worsen outcomes.

Finally, the reported results cannot be compared quantitatively because endpoint definitions differ across series. In one study, an overall success of 85.2% included eyes with residual focal scarring [3]. Success in another study required resolution of all preoperative motility restriction [2]. In a third, success was defined by the ability of the fornix to accommodate a type I keratoprosthesis [9]. These are not equivalent measurements of the same outcome. Hence, numerical differences between techniques should be interpreted as descriptive until anatomical and functional endpoints are reported separately using standardized definitions.

Limitations and Future Directions

The evidence supporting fornix reconstruction remains limited. No randomized controlled trials have evaluated graft materials, surgical techniques, or adjunctive treatments. Out of the 15 primary studies that were summarized in Table 2, only one was a comparative study. The remainder were non-comparative case series, with sample sizes ranging from 7 to 80 eyes. Several treatment comparisons are based on very small subgroups [3,8].

The studies also differ in disease etiology, severity, and especially definitions of surgical success, making reported success rates difficult to directly compare. This heterogeneity, in addition to most studies coming from a limited number of centers, limits the generalizability of findings [2,3,6]. Follow-up was generally limited to about 14-26 months, with only a small number of series reporting beyond three years. As a result, this may not accurately capture long-term progression of chronic cicatrizing disease. In addition, the reported effects of adjuncts such as MMC and anchoring sutures are based on observational associations and may be influenced by confounding by indication [3]. The review itself is further limited by reliance on a single database, restriction to English-language publications, screening by one reviewer, and the absence of a formal quality-grading instrument. Finally, some recommendations rely on evidence extrapolated from other clinical settings, including fibrin glue, OMG donor-site morbidity, alloplastic fornix stenting, intraoperative 5-FU, and rituximab [22,24,29,30,37,43,44].

To address these limitations of the fornix reconstruction literature, future research should comprise more standardized and comparative research. Studies should use a common definition of success and report anatomical and functional outcomes separately, as a deep fornix with residual motility restriction differs from recurrent symblepharon. Prospective multicenter studies using standardized severity grading would improve comparability and reduce the reliance on small, single-center studies. Longer follow-up would also provide a stronger measure of long-term durability of fornix repair [3,8,42]. Future studies could also clarify the true effects of adjuncts such as MMC and anchoring sutures, which are currently supported only by observational associations [3].

Several emerging approaches may also expand treatment options, although none has yet been evaluated with fornix-specific outcomes. Tissue engineering can create personalized conjunctival substitutes by culturing autologous limbal, conjunctival, and oral mucosal epithelial cells on scaffolds that mimic natural eye tissue. Such engineered substitutes may reduce reliance on cadaveric or allogeneic donors [45-47]. Contact lens-based delivery of expanded epithelial cells has shown potential in limbal stem cell deficiency but has not been studied for fornix reconstruction [48,49]. Finally, more targeted immunomodulation may also improve the durability of reconstruction in cicatrizing autoimmune disease such as MMP, although current evidence is limited to systemic disease control rather than fornix-specific outcomes [43,44].

## Conclusions

Fornix reconstruction restores forniceal anatomy in patients with symblepharon and conjunctival contracture. In the series reviewed, reconstruction was associated with improvements in ocular motility, ocular surface stability, and the ability to proceed to further reconstruction. The components consistently described in successful reconstructions are complete cicatrix lysis, replacement of the deficient lining in proportion to the tissue lost, secure fixation at the fornix depth, selective use of antifibrotic therapy, and control of postoperative inflammation. These principles recur across published series but remain observationally derived rather than established, and no study has isolated the contribution of any individual component. Symblepharon severity and inflammatory activity are the variables that are most frequently associated with surgical results. Durability appears poorest in progressive autoimmune disease, underscoring the need for long-term surveillance. The severity-based framework presented in this review synthesizes observational evidence and expert practice; it is not a validated treatment algorithm and should not be interpreted as a standard of care. The limitations of the evidence base are substantial. No randomized trials have been conducted; only one comparative study was identified, and definitions of surgical success vary across nearly all series. Prospective multicenter studies using standardized severity grading, prespecified anatomical and functional endpoints reported separately, and follow-up beyond three years are needed before the relative effectiveness and durability of these approaches can be established.
